# Identification through fine mapping and verification using CRISPR/Cas9-targeted mutagenesis for a minor QTL controlling grain weight in rice

**DOI:** 10.1007/s00122-020-03699-6

**Published:** 2020-10-17

**Authors:** Aye Nyein Chan, Lin-Lin Wang, Yu-Jun Zhu, Ye-Yang Fan, Jie-Yun Zhuang, Zhen-Hua Zhang

**Affiliations:** 1 State Key Laboratory of Rice Biology and Chinese National Center for Rice Improvement, China National Rice Research Institute, Hangzhou, 310006 China; 2 Lishui Institute of Agricultural and Forestry Sciences, Lishui, 323000 China; 3 Advanced Center for Agricultural Research and Education, Yezin Agricultural University, Naypyitaw, 15013 Myanmar

## Abstract

**Key message:**

A minor QTL for grain weight in rice, *qTGW1.2b*, was fine-mapped. Its casual gene *OsVQ4* was confirmed through CRISPR/Cas9-targeted mutagenesis, exhibiting an effect that was larger than the original QTL effect.

**Abstract:**

The CRISPR/Cas system exhibits a great potential for rice improvement, but the application was severely hindered due to insufficient target genes, especial the lack of validated genes underlying quantitative trait loci having small effects. In this study, a minor QTL for grain weight, *qTGW1.2b*, was fine-mapped into a 44.0 kb region using seven sets of near isogenic lines (NILs) developed from the *indica* rice cross (Zhenshan 97)^3^/Milyang 46, followed by validation of the causal gene using CRISPR/Cas9-targeted mutagenesis. In the NIL populations, 1000-grain weight of the Zhenshan 97 homozygous lines decreased by 0.9–2.0% compared with the Milyang 46 homozygous lines. A gene encoding VQ-motif protein, *OsVQ4*, was identified as the candidate gene based on parental sequence differences. The effect of *OsVQ4* was confirmed by creating CRISPR/Cas9 knockout lines, whose 1000-grain weight decreased by 2.8–9.8% compared with the wild-type transgenic line and the recipient. These results indicate that applying genome editing system could create novel alleles with large phenotypic variation at minor QTLs, which is an effective way to validate causal genes of minor QTLs. Our study establishes a strategy for cloning minor QTLs, which could also be used to identify a large number of potential target genes for the application of CRISPR/Cas system.

**Electronic supplementary material:**

The online version of this article (10.1007/s00122-020-03699-6) contains supplementary material, which is available to authorized users.

## Introduction

Rice (*Oryza sativa* L.) is one of the most important cereal crops. More than half of the global population use rice as the main food. Development of superior rice varieties is essential to ensure food security. Genetic variation is the basic resource for crop improvement, which has been greatly reduced during domestication and artificial selection (Huang et al. 2012; Wang et al. 2014). Low level of genetic diversity has become a major bottleneck for rice improvement. To enhance genetic diversity of modern rice varieties, considerable efforts have been made in two ways, i.e., introducing allelic variations from wild rice and creating novel alleles by artificial mutagenesis. Introgression of favorable wild alleles into rice varieties has been successful for insect and disease resistance (Hajjar and Hodgkin 2007; Mammadov et al. 2018), but not for grain yield. Moreover, wild resources have become less and less available because the diminishing of their natural habitats (Akimoto et al. 1999; Song et al. 2005; Xie et al. 2010). Broadening genetic variations by means of artificial mutagenesis has been widely used. According to the International Atomic Energy Agency, 833 mutant rice varieties have been officially registered (https://mvd.iaea.org). Nevertheless, mutations induced by conventional methods are random, requiring great efforts to identify favorable mutants. Over the past decade, a new genome editing technology called CRISPR/Cas9 has emerged, providing a simple and more accurate tool for creating targeted mutations (Mussolino and Cathomen 2013; Chen et al. 2019; Manghwar et al. 2019). This system has been applied to create new alleles for rice improvement (Shen et al. 2017; Huang et al. 2020; Li et al. 2020b; Zeng et al. 2020).

Available target gene is a prerequisite for applying CRISPR/Cas9. Tremendous progress has been made in rice gene cloning, but only a small proportion of casual genes for quantitative trait loci (QTLs) have been identified. Among key traits determining grain yield in rice, grain weight and size is the trait having the largest number of QTLs identified in primary mapping. More than 500 QTLs were documented in Gramene (https://www.gramene.org), distributing throughout the 12 chromosomes of rice. To date, 20 causal genes of QTLs for grain weight and size have been validated (Li et al. [Bibr CR26][Bibr CR23]; Ma et al. 2019; Wang et al. 2019a; Dong et al. 2020; Ruan et al. 2020; Shi et al. 2020), which is also higher than other yield traits. These genes were sparsely distributed on eight of the 12 rice chromosomes. Lack of validated genes underlying QTLs for yield traits has severely hindered the application of CRISPR/Cas9.

Complex traits are generally controlled by a small number of genes having large effects and a large number of genes having small effects. In a population segregating a minor-effect QTL, the respective parental alleles may each possess partial functions, thereby limiting the magnitude of observable phenotypic contrast. This makes it difficult to validate their casual genes by complementation test. Producing target gene knockout mutants using CRISPR/Cas9 and validating the gene effect by mutational analysis might be a promising approach (Zhang et al. 2016). In our previous studies, three minor QTLs for grain weight were resolved in a 4.5 Mb region on the long arm of chromosome 1, using near isogenic lines (NILs) derived from a cross between *indica* rice cultivars Zhenshan 97 (ZS97) and Milyang 46 (MY46) (Wang et al. 2015). One of them, *qTGW1.2b*, was delimited into a 418.8 kb region. In the present study, this QTL was fine-mapped into a 44.0 kb region and its casual gene was validated using CRISPR/Cas9. Our study provides and effective strategy for cloning minor QTLs in rice.

## Materials and methods

### Development of NIL populations

**Fig. 1:**
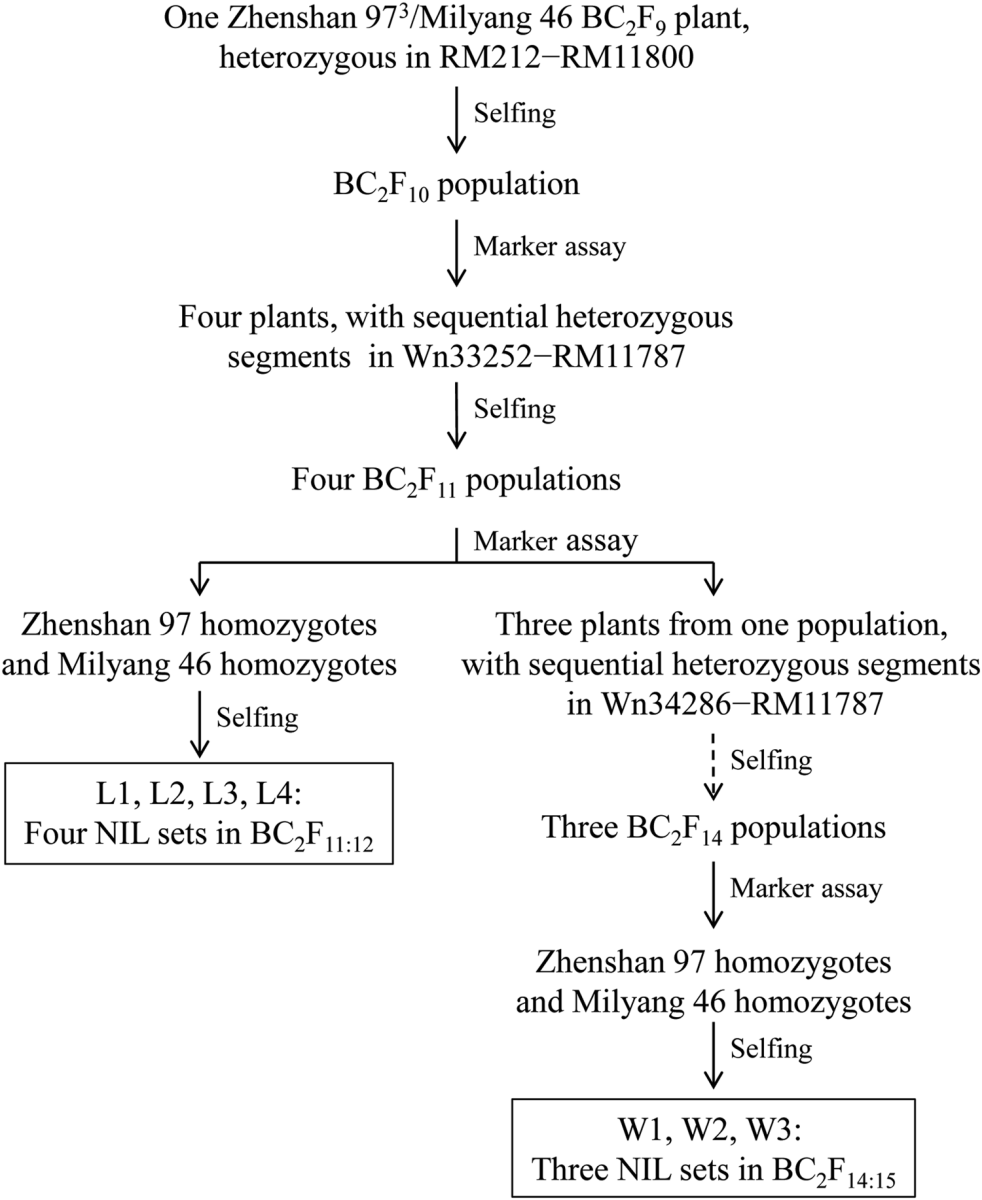
Development of the near isogenic lines used in this study

Seven NIL populations were used for QTL analysis in this study, including four populations in BC_2_F_11:12_ and three populations in BC_2_F_14:15_ (Table S1). They were derived from a BC_2_F_9_ plant of the rice cross ZS97^3^/MY46 as described below and illustrated in Fig. 1.

The BC_2_F_9_ plant was selfed to develop a BC_2_F_10_ population. Four BC_2_F_10_ plants with sequential heterozygous segments extending from Wn33252 to RM11787, were selected and selfed. In the resultant four BC_2_F_11_ populations, plants carrying homozygous alleles of a single parental type throughout the segregating region, either ZS97 or MY46 type, were identified. Selfing seeds of these plants resulted in the development of four NIL populations in BC_2_F_11:12._ The numbers of ZS97 and MY46 homozygous lines were 16 and 20 in L1, 19 and 20 in L2, 20 and 18 in L3, and 42 and 42 in L4, respectively. These populations were used for QTL mapping, and the region segregating *qTGW1.2b* was narrowed down to Wn34286–RM11787.

Then, three plants were selected from the BC_2_F_11_ population that was used to develop L4. They carried sequential heterozygous segments extending from Wn34286 to RM11787, These plants were selfed for three generations. In the resultant three BC_2_F_14_ populations, ZS97 homozygotes and MY46 homozygotes were identified and selfed. Three NIL populations in BC_2_F_14:15_ were developed. The numbers of ZS97 and MY46 homozygous lines were 36 and 38 in W1, 40 and 39 in W2, and 40 and 40 in W3, respectively.

### DNA marker analysis for population development and QTL mapping

Total DNA was extracted using 2 cm-long leaves following the method of Zheng et al. (1995). PCR procedure was conducted according to Chen et al. (1997) and the products were separated on 6% non-denaturing polyacrylamide gels and visualized using silver staining. A total of 17 polymorphic DNA markers were used for QTL mapping (Table S2). Six simple sequence repeat (SSR) markers were selected from the Gramene database (https://www.gramene.org), and 11 InDel markers were designed according to the sequences differences between ZS97 and MY46 as detected by whole genome re-sequencing and targeted next-generation sequencing.

### Sequence analysis of candidate genes

Sequence analysis was performed for two annotated genes located in the *qTGW1.2b* region, including *OsCDPK2* (*LOC_Os01g59360*) and *OsVQ4* (*LOC_Os01g59410*). DNA was extracted using DNeasy Plant Mini Kit (QIAGEN, Hilden, German) according to the manufacturer’s instruction. Primers 360s1 and 360s2 for *OsCDPK2* and 410s for *OsVQ4* (Table S2) were designed according to Nipponbare and ZS97 genomic sequences (https://rice.plantbiology.msu.edu and https://rice.hzau.edu.cn/rice). Products amplified from the genomic DNA of ZS97 and MY46 were sequenced by the Sanger method. Nucleotide sequence and the predicted amino acid sequence between ZS97 and MY46 were compared.

### Generation of knockout mutants using CRISPR/Cas9 system

The CRISPR/Cas9 system was used to generate knockout mutants for one of the annotated genes, *OsVQ4*. Two targets, located at + 30 to + 49 and + 34 to + 53 in the coding region, respectively, were selected using the web-based tool CRISPR-GE (https://skl.scau.edu.cn). The oligonucleotides 410cri-1 and 410cri-2 (Table S2) were designed and independently ligated into BGK03 vector (BIOGLE Co., Ltd, Hangzhou, China) according to the manufacturer’s instruction. The original BGK03 vector contains a rice U6 promoter for activating the target sequence, a Cas9 gene driven by the maize ubiquitin promoter, and a hygromycin marker gene driven by the *Cauliflower mosaic virus* 35S promoter.

According to the rice information gateway database (https://rice.hzau.edu.cn/rice), coding sequences of the six annotated genes in the *qTGW1.2b* region are identical between Nipponbare and ZS97, so is the promoter sequence of *OsVQ4*. Thus, Nipponbare was used as the recipient for transformation. The two constructs were separately introduced into Nipponbare using *Agrobacterium tumefaciens*-mediated transformation, which was performed by BioRun Co., Ltd (Wuhan, China). Genomic DNA of T_0_ plants was extracted using the DNeasy Plant Mini Kit. They were assayed with Hyg marker for hygromycin gene (Table S2). The *OsVQ4* gene fragment was amplified from each Hyg-positive plant using the sequencing primer 410s. The product was directly sequenced by the Sanger method and decoded using the web-based tool DSDecodeM (https://skl.scau.edu.cn/dsdecode). For cri-1, 1 bp insertion was found at 3 bp upstream from its PAM sequence. Of the 11 independent T_0_ plants tested, one showed no mutation, four were homozygous mutants, two were heterozygous mutants, and the other four were not decoded. For cri-2, 1 bp insertion was found 3 bp upstream from its PAM sequence. Of the four independent T_0_ plants tested, two were homozygous mutants and the other two were not decoded. Five of the T_0_ plants were selected and selfed, including the wild-type (WT) plant, two homozygous mutants for cri-1, and two homozygous mutants for cri-2. In each resultant T_1_ family, 16 plants were assayed with the 410m marker for detecting the 1-bp insertion (Table S2). Genotypes of the five families were confirmed.

### Field experiments and phenotyping

The rice materials were tested at the China National Rice Research Institute in Hangzhou, Zhejiang province, China. The four BC_2_F_11:12_ populations were tested in 2014, the three BC_2_F_14:15_ populations were tested in 2016, and one of the BC_2_F_14:15_ populations (W3) was further tested in 2018 and 2019. The five transgenic lines and the recipient Nipponbare were tested in 2019. A planting density of 16.7 cm × 26.7 cm was used. Field management was performed following the normal agricultural practice.

For the NIL populations, a randomized complete block design with two replications was applied. In each replication, one line was grown in a single row of eight plants. Seven traits were tested, including 1000-grain weight (TGW, g), grain length (GL, mm), grain width (GW, mm), number of spikelets per panicle (NSP), number of grains per panicle (NGP), spikelet fertility (SF), and heading date (HD, d). Of them, TGW, GL and GW were measured for all populations in all experiments, and the other four were only scored for the four populations tested in 2014. HD was recorded for individual plants and averaged for each replication. At maturity, five of middle six plants in each row were bulk-harvested and measured for the six yield traits. Of these, TGW, GL and GW were evaluated using fully filled grains following the procedure reported by Zhang et al. (2016). In short, the grains were soaked in 3.5 mol/L NaCl solution and floating grains were removed. Approximately 18 g fully filled grains were obtained and dried. They were divided into two halves and measured for TGW, GL and GW using an automatic seed counting and analyzing instrument (Model SC-G, Wanshen Ltd, Hangzhou, China).

For the five transgenic lines, each line was grown in 10 rows of 12 plants, and HD was recorded for the middle 100 plants. For Nipponbare, 20 rows of 12 plants were grown, and HD was recorded for the middle 200 plants. At maturity, 20 plants of each transgenic line and 40 plants of Nipponbare were individually harvested. They were measured for eight traits, including grain yield per plant (GY, g), number of panicles per plant (NP), NSP, NGP, SF, TGW, GL and GW. For TGW, GL and GW, approximately 6 g fully filled grains were divided into two halves and tested.

### Data analysis

For each of the NIL populations, phenotypic differences between the two genotypic groups were tested using two-way analysis of variance (ANOVA). The analysis was performed using the SAS procedure GLM (SAS Institute 1999) as described previously (Dai et al. 2008). Given the detection of a significant difference (*P* < 0.05), the same data were used to estimate the genetic effects of the QTL, including additive effect and the proportion of phenotypic variance explained (*R*^*2*^). For the transgenic materials and Nipponbare, Duncan’s Multiple Range Test was used to examine the phenotypic differences among them.

## Results

### Validation and delimitation of *qTGW1.2b* into a 108.6 kb region

Four NIL populations in BC_2_F_11:12_ were used in the first experiment that was conducted in 2014. Distributions of TGW, GL and GW in these populations are shown in Fig. S1a. Differences between ZS97 and MY46 homozygous genotypes were observed for TGW and GL in three populations (L2, L3, L4), especially in L2. Difference between the two genotypic groups was also observed for GL in L1. In all cases, the MY46 homozygous lines were clustered toward the area of the higher values, and ZS97 homozygous lines toward the lower-value area.

**Fig. 2:**
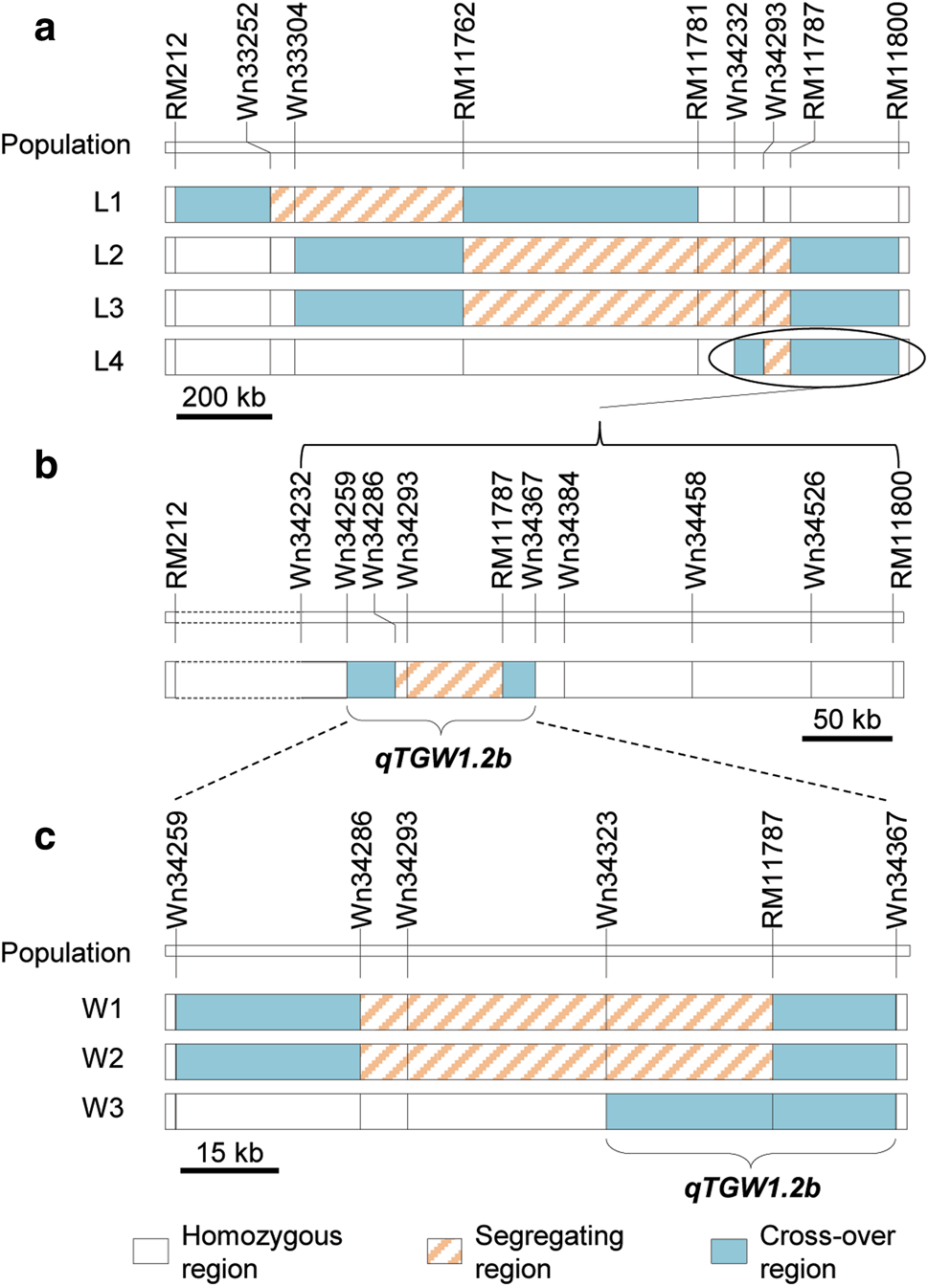
Genotypic composition of the near isogenic lines (NILs) in the target region. **a** Composition of four NIL populations in BC_2_F_11:12_. **b** Composition of the NIL population L4 updated with more polymorphic markers. **c** Composition of three NIL populations in BC_2_F_14:15_

Results of two-way ANOVA for phenotypic differences between the two genotypic groups in each population are presented in Table 1. Highly significant (*P* < 0.0001) genotypic effects on TGW and GL were detected in L2, L3 and L4, and the enhancing alleles were all derived from MY46. In L2, the additive effects were 0.30 g for TGW and 0.073 mm for GL, having *R*^*2*^ values of 29.76 and 55.55%, respectively. In L3, the additive effects were 0.27 g for TGW and 0.048 mm for GL, having *R*^*2*^ values of 30.00 and 41.29%, respectively. In L4, the additive effects were 0.16 g for TGW and 0.025 mm for GL, having *R*^*2*^ values of 11.15 and 13.12%, respectively. These results indicate that a QTL for TGW and GL, i.e., *qTGW1.2b* reported by Wang et al. (2015), was located in the common segregating region of L2, L3 and L4. As shown in Fig. 2a, this region is the interval Wn34232–RM11800, having a physical distance of 341.9 kb in the Nipponbare genome. This QTL also had small effects on NSP and NGP in L4, with the enhancing allele derived from ZS97.

**Table 1:** QTL effects detected in the four NIL populations in BC_2_F_11:12_

Name	Trait^a^	Phenotype (mean ± SD)^b^		*A*^c^	*R*^2^ (%)^d^
		NIL^ZS97^	NIL^MY46^		
L1	TGW	29.45 ± 0.21	29.50 ± 0.17	n.s	
	GL	8.577 ± 0.031	8.630 ± 0.035	0.026****	23.51
	GW	3.290 ± 0.019	3.282 ± 0.019	n.s	
	NSP	101.1 ± 6.9	101.9 ± 5.5	n.s	
	NGP	93.5 ± 6.2	94.2 ± 5.2	n.s	
	SF	92.4 ± 1.9	92.4 ± 1.3	n.s	
	HD	73.3 ± 0.6	73.0 ± 0.6	n.s	
L2	TGW	29.36 ± 0.36	29.96 ± 0.32	0.30****	29.71
	GL	8.539 ± 0.044	8.685 ± 0.045	0.073****	55.55
	GW	3.236 ± 0.028	3.228 ± 0.021	n.s	
	NSP	102.5 ± 5.5	102.2 ± 4.7	n.s	
	NGP	92.1 ± 4.8	91.4 ± 4.9	n.s	
	SF	89.8 ± 2.0	89.4 ± 2.9	n.s	
	HD	73.6 ± 0.7	73.5 ± 0.7	n.s	
L3	TGW	29.56 ± 0.31	30.10 ± 0.31	0.27****	30.00
	GL	8.562 ± 0.042	8.569 ± 0.035	0.048****	41.29
	GW	3.285 ± 0.016	3.281 ± 0.017	n.s	
	NSP	103.1 ± 3.4	104.2 ± 3.6	n.s	
	NGP	94.5 ± 3.5	95.5 ± 3.3	n.s	
	SF	91.7 ± 2.0	91.7 ± 1.5	n.s	
	HD	73.8 ± 0.7	74.1 ± 0.9	n.s	
L4	TGW	29.37 ± 0.29	29.68 ± 0.32	0.16****	11.15
	GL	8.546 ± 0.040	8.596 ± 0.048	0.025****	13.12
	GW	3.341 ± 0.021	3.318 ± 0.017	n.s	
	NSP	84.2 ± 3.7	82.2 ± 3.5	− 1.00*	2.64
	NGP	76.2 ± 3.4	74.4 ± 4.1	− 0.96*	2.31
	SF	90.6 ± 1.8	90.5 ± 2.6	n.s	
	HD	68.3 ± 0.4	68.4 ± 0.5	n.s	

^a^TGW, 1000-grain weight (g); GL, Grain length (mm); GW, grain width (mm); NSP, number of spikelets per panicle; NGP, number of grains per panicle; SF, spikelet fertility (%); HD, heading date (d)

^b^NIL^ZS97^ and NIL^MY46^ are near isogenic lines with Zhenshan 97 and Milyang 46 homozygous genotypes in the segregating region, respectively

^c^Additive effect of replacing a Zhenshan 97 allele with a Milyang 46 allele. n.s., non-significant; *P < 0.05; ****P < 0.0001

^d^Proportion of phenotypic variance explained by the QTL effect

For the remaining population tested in 2014, L1, the segregating region did not include the *qTGW1.2b* region RM11781–RM11800 reported by Wang et al. (2015) (Fig. 2a). This population was initially used as a negative control. No significant genotypic effect was detected except for GL. The additive effect was 0.026 mm and *R*^*2*^ was 23.51%. These results suggest that a QTL responsible for GL but not for TGW was linked to *qTGW1.2b*.

In the updated region of *qTGW12.b*, Wn34232–RM11800, the two cross-over regions occupied the major portion (Fig. 2a). Therefore, we developed InDel markers in the cross-over regions according to the sequence differences between ZS97 and MY46. Six polymorphic markers were selected to assay the individual lines of L4. One marker (Wn34286) was heterozygous and other five markers (Wn34259, Wn34367, Wn34384, Wn34458 and Wn34526) were homozygous (Fig. 2b). Consequently, *qTGW1.2b* was narrowed down to a 108.6 kb region flanked by Wn34259 and Wn34367.

### Fine-mapping of *qTGW1.2b*

Fine-mapping was continued using three NIL populations segregating within the updated *qTGW1.2b* region, including W1, W2 and W3 (Fig. 2c). Differences between ZS97 and MY46 homozygous genotypes were observed for TGW and GL in all the three populations (Fig. S1b). The MY46 homozygous lines were clustered toward the area of higher values, while ZS97 homozygous lines toward the lower-value area. In all the three populations, significant effects were detected on TGW and GL, but not on GW (Table 2). The additive effects ranged from 0.13 to 0.20 g for TGW and from 0.021 to 0.038 mm for GL, with *R*^*2*^ ranging as 5.05−9.26% and 14.14−29.68%, respectively. The enhancing alleles were all derived from MY46. Obviously, *qTGW1.2b* was located within the common segregating region of the three populations. As shown in Fig. 2c, this is an interval flanked by Wn34323 and Wn34367, corresponding to a 44.0 kb region in the Nipponbare genome.

**Table 2:** QTL effects detected in the three NIL populations in BC_2_F_14:15_

Name	Year	Trait^a^	Phenotype (mean ± SD)^b^		*A*^c^	*R*^2^ (%)^d^
			NIL^ZS97^	NIL^MY46^		
W1	2016	TGW	27.53 ± 0.39	27.93 ± 0.52	0.20**	9.26
		GL	8.325 ± 0.030	8.401 ± 0.047	0.038****	29.68
		GW	3.095 ± 0.018	3.093 ± 0.022	n.s	
W2	2016	TGW	25.78 ± 0.46	26.03 ± 0.43	0.13*	5.05
		GL	8.289 ± 0.045	8.340 ± 0.046	0.026****	17.87
		GW	3.040 ± 0.030	3.043 ± 0.028	n.s	
W3	2016	TGW	27.29 ± 0.34	27.54 ± 0.45	0.13**	6.30
		GL	8.352 ± 0.031	8.394 ± 0.037	0.021****	14.14
		GW	3.065 ± 0.017	3.067 ± 0.020	n.s	
	2018	TGW	28.12 ± 0.45	28.49 ± 0.57	0.19**	8.90
		GL	8.469 ± 0.046	8.528 ± 0.059	0.030****	19.36
		GW	3.071 ± 0.034	3.077 ± 0.032	n.s	
	2019	TGW	28.78 ± 0.24	29.03 ± 0.25	0.13****	12.18
		GL	8.295 ± 0.036	8.337 ± 0.033	0.020****	20.20
		GW	3.213 ± 0.018	3.208 ± 0.023	n.s	

^a^TGW, 1000-grain weight (g); GL, grain length (mm); GW, grain width (mm)

^b^NIL^ZS97^ and NIL^MY46^ are near isogenic lines with Zhenshan 97 and Milyang 46 homozygous genotypes in the segregating region, respectively

^c^Additive effect of replacing a Zhenshan 97 allele with a Milyang 46 allele. n.s., non-significant; *P < 0.05; **P < 0.01; ****P < 0.0001

^d^Proportion of phenotypic variance explained by the QTL effect

The population carrying the smallest segregating region, W3, was grown in two more years. Consistent with the previous observations, the QTL effects were significant on TGW and GL, but not on GW (Table 2). In the two years, the additive effects for TGW were 0.19 and 0.13 g, with *R*^*2*^ of 8.90 and 12.18%; and the additive effects for GL were 0.030 and 0.020 mm, with *R*^*2*^ of and 19.36 and 20.20%. Again, the enhancing alleles were all derived from MY46. These results indicate that *qTGW1.2b* has a stable effect on grain weight and size.

### Candidate gene analysis of *qTGW1.2b*

Based on the MSU Rice Genome Annotation Project Database and Resource (https://rice.plantbiology.msu.edu) and the rice genomic variation and functional annotation database of Huazhong Agricultural University (https://ricevarmap.ncpgr.cn/v2/), six annotated genes are predicted in the 44.0 kb region for *qTGW1.2b* (Table S3). Four of them encode expressed proteins with unknown function, including *LOC_Os01g59370*, *LOC_Os01g59390*, *LOC_Os01g59400* and *LOC_Os01g59420*. In addition, all of the four genes had no homolog in other plant species (https://blast.ncbi.nlm.nih.gov/blast.cgi). Of the remaining two annotated genes, *LOC_Os01g59360* encodes calcium dependent protein kinase 2 (OsCDPK2), and *LOC_Os01g59410* encodes VQ motif-containing protein 4 (OsVQ4). The calcium dependent protein kinases and VQ proteins are involved in various biological processes in plants, including growth, development, and abiotic and biotic stress responses (Wang et al. 2010; Li et al. 2014; Jing and Lin 2015; Shi et al. 2018; Zhong et al. 2018). Moreover, two VQ proteins genes, *AtVQ14 *and *OsVQ13*, have been found to control seed size in plant (Garcia et al. 2003; Wang et al. 2010; Uji et al. 2019).

Sequence comparisons of *OsCDPK2* and *OsVQ4* were conducted between full-length genomic DNA of ZS97 and MY46. For *OsCDPK2*, six single nucleotide polymorphisms (SNPs) and two 1 bp InDels were found, all of which were located in introns (Fig. S2). For *OsVQ4* that consists of only one exon, a 3 bp InDel and three SNPs were identified (Fig. S3). The InDel resulted in an additional aspartic acid at residue 157 in MY46 compared with ZS97 (Fig. S4). One SNP (G259A) resulted in the substitution of glycine in ZS97 to serine in MY46 at residue 86. The remaining two SNPs were synonymous mutations. These results suggest that *OsVQ4* was likely to be a candidate gene for *qTGW1.2b*.

### Knockout of *OsVQ4* using the CRISPR/Cas9 system

**Fig. 3:**
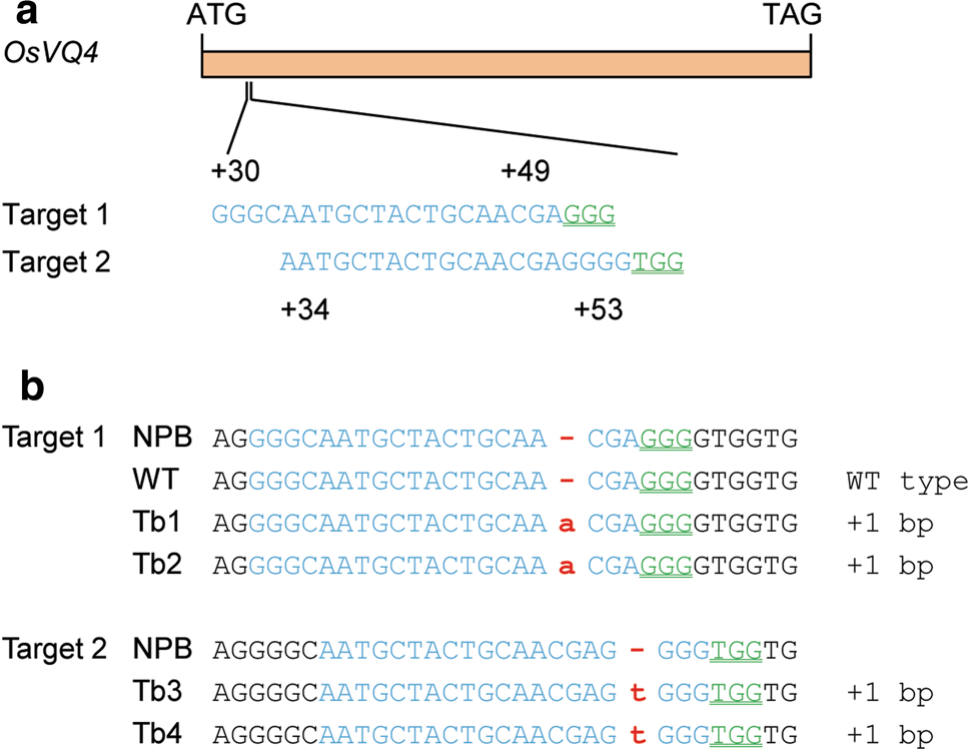
Knockout of *OsVQ4* using CRISPR/Cas9 system. **a** Schematic of *OsVQ4* gene structure and the CRISPR/Cas9 target site. *OsVQ4* contains a single exon indicated by orange rectangles. The translation initiation codon (ATG) and termination codon (TAG) are shown. The target site nucleotides are shown in blue letters. The protospacer adjacent motif (PAM) site is marked in green and underlined. **b** Sequence of Nipponbare (NPB), the wild-type transgenic line (WT) and the four mutational lines of *OsVQ4* in the target region. Insertions are indicated by red letters

The CRISPR/Cas9 system was used to generate knockout mutants for OsVQ4 (Fig. 3a). Four independent mutational lines were selected to investigate the effects of OsVQ4, including Tb1 and Tb2 for cri-1, and Tb3 and Tb4 for cri-2 (Fig. 3b). The recipient Nipponbare and one WT-type transgenic line were used as the controls. Nine traits were measured, including TGW, GL, GW, NP, NSP, NGP, SF, GY and HD. Duncan’s Multiple Range Test was performed to determine the phenotypic differences among the six lines.

For TGW and GL, no significant difference was found between Nipponbare and the WT-type transgenic line, but significant reductions were observed in all the four mutational lines (Table 3). Compared with Nipponbare, the mutational lines were smaller by 4.9–9.8% for TGW and 1.5–3.5% for GL. Compared with the WT line, the mutational lines were smaller by 2.8–7.9% for TGW and 1.5–3.5% for GL. For GW, the effects were inconsistent. Compared with Nipponbare, the WT line showed significant increase, three mutational lines showed significant reductions, and one mutational line showed no significant difference. These results were in agreement with the effect detected for *qTGW1.2b*, showing that *qTGW1.2b/OsVQ*4 were responsible for controlling grain weight and grain length.

**Table 3:** Grain size traits in Nipponbare (NPB), the wild-type transgenic line (WT) and the four mutational lines of OsVQ4

Line	1000-grain weight (g)			Grain length (mm)			Grain width (mm)		
	Mean ± SD^a^	D1^b^	D2^c^	Mean ± SD	D1	D2	Mean ± SD	D1	D2
NPB	25.79 ± 0.71a			7.303 ± 0.120a			3.176 ± 0.044b		
WT	25.24 ± 1.64a	− 2.1		7.301 ± 0.178a	0.0		3.246 ± 0.056a	2.2	
Tb1	23.48 ± 0.98cd	− 9.0	− 7.0	7.123 ± 0.102c	− 2.5	− 2.4	3.056 ± 0.067d	− 3.8	− 5.9
Tb2	23.25 ± 0.89d	− 9.8	− 7.9	7.195 ± 0.091b	− 1.5	− 1.5	3.064 ± 0.054cd	− 3.5	− 5.6
Tb3	24.53 ± 0.93b	− 4.9	− 2.8	7.044 ± 0.092d	− 3.5	− 3.5	3.163 ± 0.039b	− 0.4	− 2.6
Tb4	24.02 ± 0.60bc	− 6.9	− 4.8	7.121 ± 0.087c	− 2.5	− 2.5	3.093 ± 0.035c	− 2.6	− 4.7

^a^Numbers with different letters are significantly different at *P* < 0.05 based on Duncan’s multiple range tests (NPB, 40 plants. WT and mutational lines, 20 plants)

^b^D1, increase over NPB (%)

^c^D2, increase over WT (%)

Among the other five yield traits, significant differences were detected for NSP, NGP and SF, but not for NP and GY (Table S4). For NSP, significant increase was observed in all the four mutational lines, larger by 13.4–19.2% than Nipponbare, and by 15.1–21.0% than WT. For NGP, significant increase was observed in two mutational lines, Tb2 and Tb4, larger by 12.3 and 15.3% than Nipponbare, and by 13.7 and 16.8% than WT. For SF, significant reduction was observed in one mutational line, Tb3, lower by 13.7% than Nipponbare and 16.8% than WT. For HD, significant reductions were observed in the four mutational lines than WT, although the differences were low as 1.0–2.3 d or 1.5–2.9%. No obvious change was observed for plant stature and health in the mutational lines (Fig. S5).

## Discussion

Most important agronomic traits are controlled by a few QTLs having large effects and many QTLs having small effects. Among rice QTLs for which the casual genes have been validated, major QTLs accounted for the vast majority (Ashikari et al. 2005; Hori et al. 2016; Huo et al. 2017; Li et al. 2019; Ma et al. 2019; Wang et al. 2019a; Dong et al. 2020; Ruan et al. 2020; Shi et al. 2020). Only a small number of genes underlying minor QTLs for HD were validated to date (Hori et al. 2016; Chen et al. 2018). In the present study, a minor QTL for grain weight, *qTGW1.2b*, was fine-mapped using NIL populations derived from sequential residual heterozygotes. Its casual gene *OsVQ4* was validated using CRISPR/Cas9-targeted mutagenesis. In the NIL populations, TGW decreased by 0.9–2.0% in ZS97 homozygous lines compared with MY46 homozygous lines. In the knockout lines, TGW decreased by 2.8–9.8% compared with the wild-type transgenic line and the recipient. Our study establishes a strategy for cloning minor QTLs, which could also be used to identify a large number of potential target genes for the application of CRISPR/Cas system.

One inspiration from the present study was that minor QTLs could be used as new targets for enhancing genetic diversity of rice varieties. This may be done by searching for rare alleles presented in germplasm resources or by creating new alleles using artificial mutagenesis. Based on the data documented in the rice genomic variation and functional annotation database of Huazhong Agricultural University (https://ricevarmap.ncpgr.cn/v2/), two non-synonymous SNPs were found in coding region of *qTGW1.2b/OsVQ4* in 1782 varieties. One was G4A, where both ZS97 and MY46 carried G. Another was G259A, which is identical to the variation detected between ZS97 and MY46. Three haplotypes could be classified based on the two SNPs. Among them, G^4^G^259^ of the ZS97-type accounted for the highest proportion (67.7%), followed by G^4^A^259^ of the MY46-type (31.4%). The remaining A^4^A^259^ type only accounted for 0.9%. Genetic effects of the rare allele A^4^A^259^ could be tested and its value for breeding application could be evaluated. Artificial insertional mutants including an activation tagging line for *OsVQ4* were also found in the Rice Functional Genomic Express Database (https://signal.salk.edu/cgi-bin/RiceGE). These insertional mutants could also be tested and evaluated.

Loss-of-function allele of *qTGW1.2b* was produced using CRISPR/Cas9 in the present study. Although grain weight was reduced in these mutational lines (Table 3), grain yield remained stable due to compensation in grain number (Table S4). Moreover, a slight reduction was observed in heading date. Thus, the loss-of-function allele of *qTGW1.2b/OsVQ4* could be used to shorten growth period without yield penalty. Generally, the agronomic traits are determined by both positive and negative regulators. Among the 20 validated genes underlying QTLs for grain weight, eight were negative regulators, including *GW2*, *TGW2*, *GS3*, *SG3*, *GL3.1/qGL3*, *TGW3/qTGW3/GL3.3*, *GSE5* and *TGW6* (Li et al. [Bibr CR26][Bibr CR23]; Ruan et al. 2020). Loss function of these genes results in increase of grain weight and grain yield. In our previous studies, eight minor QTLs for grain weight and size were fine-mapped (Zhang et al. 2016,2020; Dong et al. 2018; Wang et al. 2019b; Zhu et al. 2019). There is a high probability that negative regulators could be identified from these minor QTLs. Applying CRISPR/Cas9 to create their loss-of-function alleles may achieve improvement in grain yield.

**Fig. 4:**
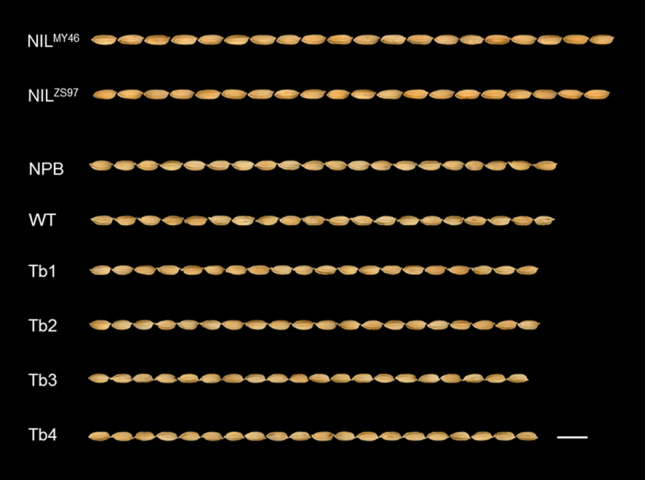
Grains of near isogenic lines (NILs), Nipponbare (NPB), the wild-type transgenic line (WT) and the four mutational lines of *OsVQ4*. ZS97: Zhenshan 97; MY46: Milyang 46. Bar = 10 mm

The VQ protein are a class of plant-specific proteins, which were found to regulate diverse plant growth and developmental processes (Wang et al. 2010; Li et al. 2014; Jing and Lin 2015; Ye et al. 2016; Lei et al. 2017). The characteristic structure of VQ proteins is the conserved VQ motif, which possesses the core sequence FxxhVQxhTG (x refers to any amino acids and h refers to hydrophobic amino acids) (Jing and Lin 2015). The primary structure of VQ proteins is highly diverse in regions other than the VQ motif (Jing and Lin 2015). Two VQ proteins, AtVQ14 and OsVQ13 were reported to control seed development in plant (Wang et al. 2010; Uji et al. 2019). In the present study, we found OsVQ4 was also responsible for grain size. Similarity of these three proteins is weak. The OsVQ4 had only 20.1% amino acid residuals identity to AtVQ14 (37/184) and 25.0% identity to OsVQ13 (46/184) (data not shown). They were classified into three different groups based on the VQ motif (Kim et al. 2013). Both *AtVQ14* and *OsVQ13* were found to function as a positive regulator for seed size. Mutation of *AtVQ14* exhibited small seed compared with wild type (Wang et al. 2010), while over-expression of *OsVQ13* produced larger grain compared with wild type (Uji et al. 2019). Our results showed that knockout of *OsVQ4* also resulted in small grain (Fig. 4). These results suggested that VQ proteins may have conserved roles in seed size, though they had highly diverse protein sequence.

Site-directed mutagenesis revealed that the function of VQ protein largely depended on its VQ-motif. Transgenes harboring mutations in the VQ motif of *AtVQ14* resulted in small seed phenotype, but transgenes harboring mutations in other regions did not had such an effect (Wang et al. 2010). This suggests that mutations in other regions had limited influence on it molecular function. The non-synonymous variations between ZS97 and MY46 were located at residues 86 and 157 in OsVQ4 protein, which were outside the VQ motif (Fig. S4). Twenty-seven homologs of OsVQ4 were found in gramineae using BLASTP program from National Center for Biotechnology Information with a *E* value < 10^–5^ (https://blast.ncbi.nlm.nih.gov/blast.cgi). At residue 86, seven homologs carried glycine, seven carried serine, and the other 13 had deletion. At the region harboring residue 157, substitutions, insertions and deletions were observed. The variation between ZS97 and MY46 occurred in non-conserved regions outside the VQ motif, which may be the reason why *qTGW1.2b* exhibited a small effect. The VQ protein functions as transcriptional regulators in plant, which usually work in combination with the WRKY transcription factors (Lai et al. 2011; Chi et al. 2013; Jing and Lin 2015; Ye et al. 2016; Lei et al. 2017). The regulation of seed size by AtVQ14 was achieved by forming a complex with a WRKY transcription factor MINI3 (Wang et al. 2010). Hence, WRKY transcription factors would be a good starting point to investigate the molecular mechanisms underlying the role of *qTGW1.2b/OsVQ4* in regulating grain size in rice.

Grain weight and grain number are two key traits determining grain yield in rice, however, the negative correlation between these two traits were frequently observed. In the present study, *qTGW1.2b* was found to have opposite effects on grain weight and number in the NIL populations (Table 1). This was also confirmed in the knockout lines, which showed smaller but more grains compared with WT (Table 3). These results suggest that *qTGW1.2b* may coordinate the trade-off between grain weight and number in rice. More recently, a mitogen-activated protein kinase (MAPK) cascade, consisting of OsMKKK10*,* OsMKK4 and OsMPK6, was found to contribute the trade-off between grain weight and number in rice, by integrating localized cell differentiation and proliferation (Guo et al. 2018, 2020; Xu et al. 2018). Knockout of *OsMKKK10* or *OsMKK4*, and knockdown of *OsMPK6*, all resulted in smaller but more grains (Guo et al. 2018; Xu et al. 2018). Members of the MAPK cascade could interact with VQ proteins to form a trimeric complex with WRKY transcription factors (Andreasson et al. 2005; Pecher et al., 2014). Investigating whether OsVQ4 interacts with the MAPK cascade will help to reveal the molecular mechanism coordinating the trade-off between grain weight and number in rice.

## Electronic supplementary material

Below is the link to the electronic supplementary material.

Supplementary file1 (DOCX 31 kb)

Supplementary file2 (PDF 161 kb)

## Data Availability

The datasets generated during the current study are available from the corresponding author on reasonable request.

## Code availability

No applicable.

## References

[CR1] Akimoto M, Shimamoto Y, Morishima H (1999). The extinction of genetic resources of Asian wild rice, *Oryza rufipogon* Griff.: a case study in Thailand. Genet Resour Crop Evol.

[CR2] Andreasson E, Jenkins T, Brodersen P, Thorgrimsen S, Petersen NHT, Zhu S, Qiu JL, Micheelsen P, Rocher A, Petersen M, Newman MA, Nielsen HB, Hirt H, Somssich I, Mattsson O, Mundy J (2005). The MAP kinase substrate MKS1 is a regulator of plant defense responses. EMBO J.

[CR3] Ashikari M, Sakakibara H, Lin S, Yamamoto T, Takashi T, Nishimura A, Angeles ER, Qian Q, Kitano H, Matsuoka M (2005). Cytokinin oxidase regulates rice grain production. Science.

[CR4] Chen X, Temnykh S, Xu Y, Cho YG, McCouch SR (1997). Development of a microsatellite framework map providing genome-wide coverage in rice (*Oryza sativa* L). Theor Appl Genet.

[CR5] Chen K, Wang Y, Zhang R, Zhang H, Gao C (2019). CRISPR/Cas genome editing and precision plant breeding in agriculture. Annu Rev Plant Biol.

[CR6] Chen J-Y, Zhang H-W, Zhang H-L, Ying J-Z, Ma L-Y, Zhuang J-Y (2018). Natural variation at *qHD1* affects heading date acceleration at high temperatures with pleiotropism for yield traits in rice. BMC Plant Biol.

[CR7] Chi Y, Yang Y, Zhou Y, Zhou J, FanYuChen BJQZ (2013). Protein-protein interactions in the regulation of WRKY transcription factors. Mol Plant.

[CR8] Dai W-M, Zhang K-Q, Wu J-R, Wang L, Duan B-W, Zheng K-L, Cai R, Zhuang J-Y (2008). Validating a segment on the short arm of chromosome 6 responsible for genetic variation in the hull silicon content and yield traits of rice. Euphytica.

[CR9] Dong N, Sun Y, Guo T, Shi CL, Zhang YM, Kan Y, Xiang YH, Zhang H, Yang YB, Li YC (2020). UDP-glucosyltransferase regulates grain size and abiotic stress tolerance associated with metabolic flux redirection in rice. Nat Commun.

[CR10] Dong Q, Zhang Z-H, Wang L-L, Zhu Y-J, Fan Y-Y, Mou T-M, Ma L-Y, Zhuang J-Y (2018). Dissection and fine-mapping of two QTL for grain size linked in a 460 kb region on chromosome 1 of rice. Rice.

[CR11] Garcia D, Saingery V, Chambrier P, Mayer U, Jurgens G, Berger F (2003). Arabidopsis *haiju* mutants reveal new controls of seed size by endosperm. Plant Physiol.

[CR12] Guo T, Chen K, Dong NQ, Shi CL, Ye WW, Gao JP, Shan JX, Lin HX (2018). *GRAIN SIZE AND NUMBER1* negatively regulates the OsMKKK10-OsMKK4-OsMPK6 cascade to coordinate the trade-off between grain number per panicle and grain size in rice. Plant Cell.

[CR13] Guo T, Lu ZQ, Shan JX, Ye WW, Dong NQ, Lin HX (2020). *ERECTA1* acts upstream of the OsMKK10-OsMKK4-OsMPK6 cascade to control spikelet number by regulating cytokinin metabolism in rice. Plant Cell.

[CR14] Hajjar R, Hodgkin T (2007). The use of wild relatives in crop improvement: a survey of developments over the last 20 years. Euphytica.

[CR15] Hori K, Matsubara K, Yano M (2016). Genetic control of flowering time in rice: integration of Mendelian genetics and genomics. Theor Appl Genet.

[CR16] Huang X, Kurata N, Wei X, Wang ZX, Wang A, Zhao Q, Zhao Y, Liu K, Lu H, Li W (2012). A map of rice genome variation reveals the origin of cultivated rice. Nature.

[CR17] Huang L, Li Q, Zhang C, Chu R, Gu Z, Tan H, Zhao D, Fan X, Liu Q (2020). Creating novel *Wx* alleles with fine-tuned amylose levels and improved grain quality in rice by promoter editing using CRISPR/Cas9 system. Plant Biotechnol J.

[CR18] Huo X, Wu S, Zhu Z, Liu F, Fu Y, Cai H, Sun X, Gu P, Xie D, Tan L, Sun C (2017). *NOG1* increases grain production in rice. Nat Commun.

[CR19] Jing Y, Lin R (2015). The VQ motif-containing protein family of plant-specific transcriptional regulators. Plant Physiol.

[CR20] Kim DY, Kwon SI, Choi C, Lee H, Ahn I, Park SR, Bae SC, Lee SC, Hwang DJ (2013). Expression analysis of rice VQ genes in response to biotic and abiotic stresses. Gene.

[CR21] Lai Z, Li Y, Wang F, Cheng Y, Fan B, Yu JQ, Chen Z (2011). *Arabidopsis* sigma factor binding proteins are activators of the WRKY33 transcription factor in plant defense. Plant Cell.

[CR22] Lei R, Li X, Ma Z, Lv Y, Hu Y, Yu D (2017). Arabidopsis WRKY2 and WRKY34 transcription factors interact with VQ20 protein to modulate pollen development and function. Plant J.

[CR23] Li Q, Lu L, Liu H, Bai X, Zhou X, Wu B, Yuan M, Yang L, Xing Y (2020). A minor QTL, *SG3*, encoding an R2R3-MYB protein, negatively controls grain length in rice. Theor Appl Genet.

[CR24] Li N, Li X, Xiao J, Wang S (2014). Comprehensive analysis of VQ motif-containing gene expression in rice defense responses to three pathogens. Plant Cell Rep.

[CR25] Li C, Li W, Zhou Z, Chen H, Xie C, Lin Y (2020). A new rice breeding method: CRISPR/Cas9 system editing of the *Xa13* promoter to cultivate transgene-free bacterial blight-resistant rice. Plant Biotechnol J.

[CR26] Li N, Xu R, Li Y (2019). Molecular networks of seed size control in plants. Annu Rev Plant Biol.

[CR27] Ma X, Feng F, Zhang Y, Elesawi IE, Xu K, Li T, Mei H, Liu H, Gao N, Chen C, Luo L, Yu S (2019). A novel rice grain size gene *OsSNB* was identified by genome-wide association study in natural population. PLoS Genet.

[CR28] Mammadov J, Buyyarapu R, Guttikonda SK, Parliament K, Abdurakhmonov IY, Kumpatla SP (2018). Wild relatives of maize, rice, cotton, and soybean: treasure troves for tolerance to biotic and abiotic stresses. Front Plant Sci.

[CR29] Manghwar H, Lindsey K, Zhang X, Jin S (2019). CRISPR/Cas System: recent advances and future prospects for genome editing. Trends Plant Sci.

[CR30] Mussolino C, Cathomen T (2013). RNA guides genome engineering. Nat Biotechnol.

[CR31] Pecher P, Eschen-Lippold L, Herklotz S, Kuhle K, Naumann K, Bethke G, Uhrig J, Weyhe M, Scheel D, Lee J (2014). The *Arabidopsis thaliana* mitogen-activated protein kinases MPK3 and MPK6 target a subclass of ‘VQ-motif’-containing proteins to regulate immune responses. New Phytol.

[CR32] Ruan B, Shang L, Zhang B, Hu J, Wang Y, Lin H, Zhang A, Liu C, Peng Y, Zhu L (2020). Natural variation in the promoter of *TGW2* determines grain width and weight in rice. New Phytol.

[CR33] Shen L, Hua Y, Fu Y, Li J, Liu Q, Jiao X, Xin G, Wang J, Wang X, Yan C, Wang K (2017). Rapid generation of genetic diversity by multiplex CRISPR/Cas9 genome editing in rice. Sci China Life Sci.

[CR34] Shi CL, Dong NQ, Guo T, Ye WW, Shan JX, Lin HX (2020). A quantitative trait locus *GW6* controls rice grain size and yield through the gibberellin pathway. Plant J.

[CR35] Shi S, Li S, Asim M, Mao J, Xu D, Ullah Z, Liu G, Wang Q, Liu H (2018). The *Arabidopsis* calcium-dependent protein kinases (CDPKs) and their roles in plant growth regulation and abiotic stress responses. Int J Mol Sci.

[CR36] Song Z, Li B, Chen J, Lu BR (2005). Genetic diversity and conservation of common wild rice (*Oryza rufipogon*) in China. Plant Species Biol.

[CR37] Uji Y, Kashihara K, Kiyama H, Mochizuki S, Akimitsu K, Gomi K (2019). Jasmonic acid-induced VQ-motif-containing protein OsVQ13 influences the OsWRKY45 signaling pathway and grain size by associating with OsMPK6 in rice. Int J Mol Sci.

[CR39] Wang A, Garcia D, Zhang H, Feng K, Chaudhury A, Berger F, Peacock WJ, Dennis ES, Luo M (2010). The VQ motif protein IKU1 regulates endosperm growth and seed size in Arabidopsis. Plant J.

[CR38] Wang L-L, Chen Y-Y, Guo L, Zhang H-W, Fan Y-Y, Zhuang J-Y (2015). Dissection of *qTGW1.2* to three QTLs for grain weight and grain size in rice (*Oryza sativa* L.). Euphytica.

[CR40] Wang A, Hou Q, Si L, Huang X, Luo J, Lu D, Zhu J, Shangguan Y, Miao J, Xie Y (2019). The PLATZ transcription factor GL6 affects grain length and number in rice. Plant Physiol.

[CR41] Wang WH, Wang LL, Zhu YJ, Fan YY, Zhuang JY (2019). Fine-mapping of *qTGW1.2a*, a quantitative trait locus for 1000-grain weight in rice. Rice Sci.

[CR42] Wang M, Yu Y, Haberer G, Reddy P, Fan C, Goicoechea JL, Zuccolo A, Song X, Kudrna D, Ammiraju JSS (2014). The genome sequence of African rice (*Oryza glaberrima*) and evidence for independent domestication. Nat Genet.

[CR43] Xie J, Agrama HA, Kong D, Zhuang J, Hu B, Wan Y, Yan W (2010). Genetic diversity associated with conservation of endangered Dongxiang wild rice (*Oryza rufipogon*). Genet Resour Crop Evol.

[CR44] Xu R, Duan P, Yu H, Zhou Z, Zhang B, Wang R, Li J, Zhang G, Zhuang S, Lyu J, Li N, Chai T, Tian Z, Yao S, Li Y (2018). Control of grain size and weight by the OsMKKK10-OsMKK4-OsMAPK6 signaling pathway in rice. Mol Plant.

[CR45] Ye YJ, Xiao YY, Han YC, Shan W, Fan ZQ, Xu QG, Kuang JF, Lu WJ, Lakshmanan P, Chen JY (2016). Banana fruit VQ motif-containing protein5 represses cold-responsive transcription factor MaWRKY26 involved in the regulation of JA biosynthetic genes. Sci Rep.

[CR46] Zeng D, Liu T, Ma X, Wang B, Zheng Z, Zhang Y, Xie X, Yang B, Zhao Z, Zhu Q, Liu YG (2020). Quantitative regulation of waxy expression by CRISPR/Cas9-based promoter and 5'UTR-intron editing improves grain quality in rice. Plant Biotechnol J.

[CR47] Zhang H-W, Fan Y-Y, Zhu Y-J, Chen J-Y, Yu S-B, Zhuang J-Y (2016). Dissection of the *qTGW1.1* region into two tightly-linked minor QTLs having stable effects for grain weight in rice. BMC Genet.

[CR48] Zhang H, Zhu Y-J, Zhu A-D, Fan Y-Y, Huang T-X, Zhang J-F, Xie H-A, Zhuang J-Y (2020). Fine-mapping of *qTGW2*, a quantitative trait locus for grain weight in rice (*Oryza sativa* L.). PeerJ.

[CR49] Zheng KL, Huang N, Bennett J, Khush GS (1995) PCR-based marker-assisted selection in rice breeding: IRRI discussion paper series No. 12. Los Banos: international rice research institute

[CR50] Zhong Y, Guo C, Chu JJ, Liu H, Cheng ZM (2018). Microevolution of the VQ gene family in six *Fragaria* species. Genome.

[CR51] Zhu Y-J, Sun Z-C, Niu X-J, Ying J-Z, Fan Y-Y, Mou T-M, Tang S-Q, Zhuang J-Y (2019). Dissection of three quantitative trait loci for grain size on the long arm of chromosome 10 in rice (*Oryza sativa* L.). PeerJ.

