# Supplementary material for: Identification through fine mapping and verification using CRISPR/Cas9-targeted mutagenesis for a minor QTL controlling grain weight in rice
Source: Theor Appl Genet. 2020 Oct 17;134(1):327–37. doi: 10.1007/s00122-020-03699-6 (PMC7813696; doi:10.1007/s00122-020-03699-6)
Supplement: Supplementary file 1 — Supplementary file1 (DOCX 31 kb) [file 122_2020_3699_MOESM1_ESM.docx]

**Table S1** Near isogenic lines used for QTL mapping

| Year | Population | Generation | Number of lines ^a^ | | Trait ^b^ |
| --- | --- | --- | --- | --- | --- |
| of trial | name |  | NIL^ZS97^ | NIL^MY46^ |  |
| 2014 | L1 | BC_2_F_11:12_ | 16 | 20 | TGW, GL, GW, NSP, NGP, SF, HD |
|  | L2 | BC_2_F_11:12_ | 19 | 20 | TGW, GL, GW, NSP, NGP, SF, HD |
|  | L3 | BC_2_F_11:12_ | 20 | 18 | TGW, GL, GW, NSP, NGP, SF, HD |
|  | L4 | BC_2_F_11:12_ | 42 | 42 | TGW, GL, GW, NSP, NGP, SF, HD |
| 2016 | W1 | BC_2_F_14:15_ | 36 | 38 | TGW, GL, GW |
|  | W2 | BC_2_F_14:15_ | 40 | 39 | TGW, GL, GW |
|  | W3 | BC_2_F_14:15_ | 40 | 40 | TGW, GL, GW |
| 2018 | W3 | BC_2_F_14:15_ | 40 | 40 | TGW, GL, GW |
| 2019 | W3 | BC_2_F_14:15_ | 40 | 40 | TGW, GL, GW |

^a^ NIL^ZS97^ and NIL^MY46^ are near isogenic lines with Zhenshan 97 and Milyang 46 homozygous genotypes in the segregating region, respectively

^b^ TGW, 1000-grain weight (g); GL, grain length (mm); GW, grain width (mm); NSP, number of spikelets per panicle; NGP, number of grains per panicle; SF, spikelet fertility (%); HD, heading date (d)

**Table S2** Primers used in this study

| Name | Type | Forward primer (5'−3') | Reverse primer (5'−3') | Purpose |
| --- | --- | --- | --- | --- |
| RM212 | SSR | CCACTTTCAGCTACTACCAG | CACCCATTTGTCTCTCATTATG | QTL mapping |
| Wn33252 | InDel | GCATGTATCAAAGATTCGATGAGA | TGAAAACTCATGGCTACGCT | QTL mapping |
| Wn33304 | InDel | ATCCAAAATAGTTGAGGCAT | TTATTTAGCTAGATATTATAGGCTGG | QTL mapping |
| RM11762 | SSR | AAGCGACAACTGAAGGAAACTCG | TTTGAAAGTCCACTGCCAAGTGC | QTL mapping |
| RM11772 | SSR | GAAGCTAAGGTCTGGGAGAAACC | AATGGCCTTAACCAAGTAGGATGG | QTL mapping |
| RM11781 | SSR | TGCCTGATGGATAGCTGATGACC | AAGCCAACACGCACACATGC | QTL mapping |
| Wn34232 | InDel | TAGTTTTGGATAAATTCGTCCACA | TCCCTTCTCTATCAATATATAAGGCAA | QTL mapping |
| Wn34259 | InDel | AATTGAAACGATGTGACGGTAA | TTTATTCAATAAAGTTGACGGG | QTL mapping |
| Wn34286 | InDel | CAATGTTCAATGATCCGTGGTAGC | TCGTACTAGCCCTATATATTTATTGTCG | QTL mapping |
| Wn34293 | InDel | CTGAAATTGCTGGTTTAAGAGGACACA | ACGTGAGATTTCAGATATGTCAGAAGT | QTL mapping |
| Wn34323 | InDel | ACACTATTTGCAGGGGTGTT | TGAGAATGTGATGCTACAGTAAACTT | QTL mapping |
| RM11787 | SSR | GTCACCCTCCTTCTACATAGAGC | TACATAACGCAACAGACAGACG | QTL mapping |
| Wn34367 | InDel | TGGAGAGGGCGTCGACAGTGAAC | ACCGGCGCCATCTTCATCAAGCTCCAC | QTL mapping |
| Wn34384 | InDel | CCTTTCCTTTTTTCTTACGCTA | GTCGGTAGATAAATAATTAACTCCCATT | QTL mapping |
| Wn34458 | InDel | CAAAACATATATTCCCTCCGTCCT | TGATAAGAGAATATTGGCGTAGAAGT | QTL mapping |
| Wn34526 | InDel | TCATCATCTGTTCGTGGGTT | TTGAAATATAAACTCTATACAATCTGCT | QTL mapping |
| RM11800 | SSR | GTCTGTTGTCTTTGTTGCCAAGG | CTTCCTGTGCTTACTCTTCTTCTTGC | QTL mapping |
| 360s1 | - | CCGTACACCACCCGACGAA | GGGGAAACAAAATCACAGACCCT | Sequencing |
| 360s2 | - | TCATGAACAGGCTCAAGCAG | GCTGACATCAACACGATACCTT | Sequencing |
| 410s | - | CCCAAGTCCCACAGCGAAC | TCCGTCCACAGTACCATACACA | Sequencing |
| 410cri-1 | - | TGTGTGAATGCTACTGCAACGAGGGG | AAACCCCCTCGTTGCAGTAGCATTCA | Vector construction |
| 410cri-2 | - | TGTGTGGGGCAATGCTACTGCAACGA | AAACTCGTTGCAGTAGCATTGCCCCA | Vector construction |
| Hyg | - | GTTTATCGGCACTTTGCATCG | GGAGCATATACGCCCGGAGT | Knockout plant analysis |
| 410m | - | TGGTGGTGGTGGTGGTGT | CCTTGATCGGCTTCTTCCC | Knockout plant analysis |

**Table S3** Annotated genes in the *qTGW1.2b* region

| Locus ID | Physical position ^a^ (bp) | |  |  | Product |
| --- | --- | --- | --- | --- | --- |
|  | Start (5’UTR) | Start (CDS) | End (CDS) | End (3’UTR) |  |
| *LOC_Os01g59360* | 34,325,310 | 34,325,426 | 34,327,699 | 34,328,118 | Calcium dependent protein kinase 2 |
| *LOC_Os01g59370* | - | 34,337,371 | 34,336,293 | - | Expressed protein |
| *LOC_Os01g59390* | - | 34,344,620 | 34,343,540 | - | Expressed protein |
| *LOC_Os01g59400* | - | 34,351,859 | 34,351,380 | - | Expressed protein |
| *LOC_Os01g59410* | 34,356,640 | 34,356,896 | 34,357,450 | - | VQ motif-containing protein 4 |
| *LOC_Os01g59420* | 34,364,097 | 34,366,663 | 34,367,193 | 34,367,572 | Expressed protein |

^a^ - indicates absence of 5’UTR or 3’UTR

**Table S4** Six agronomic traits in Nipponbare (NPB), the wild-type transgenic line (WT) and the four mutational lines of *OsVQ4*

| Line | Number of panicles per plant | | |  | Number of spikelets per panicle | | |  | Number of grains per panicle | | |  | Spikelet fertility (%) | | |  | Grain yield (g) | | |  | Heading date (d) | | |
| --- | --- | --- | --- | --- | --- | --- | --- | --- | --- | --- | --- | --- | --- | --- | --- | --- | --- | --- | --- | --- | --- | --- | --- |
|  | Mean±SD ^a^ | D1 ^b^ | D2 ^c^ |  | Mean±SD | D1 | D2 |  | Mean±SD | D1 | D2 |  | Mean±SD | D1 | D2 |  | Mean±SD | D1 | D2 |  | Mean±SD | D1 | D2 |
| NPB | 13.2±4.6a |  |  |  | 99.6±14.3b |  |  |  | 85.7±14.7c |  |  |  | 85.8±4.4a |  |  |  | 27.30±11.99a |  |  |  | 77.7±2.1b |  |  |
| WT | 13.9±3.7a | 5.3 |  |  | 98.1±12.3b | -1.5 |  |  | 84.6±12.1c | -1.3 |  |  | 86.1±3.1a | 0.5 |  |  | 26.20±9.35a | -4.0 |  |  | 79.0±1.7a | 1.7 |  |
| Tb1 | 13.2±2.9a | 0.0 | -5.0 |  | 114.1±11.6a | 14.6 | 16.3 |  | 93.3±11.5abc | 8.9 | 10.3 |  | 81.9±6.8a | -4.4 | -4.9 |  | 26.67±7.46a | -2.3 | 1.8 |  | 77.8±1.6b | 0.1 | -1.5 |
| Tb2 | 14.8±4.8a | 12.1 | 6.5 |  | 112.9±12.5a | 13.4 | 15.1 |  | 96.2±8.0ab | 12.3 | 13.7 |  | 85.6±4.8a | -0.1 | -0.6 |  | 29.38±9.67a | 7.6 | 12.1 |  | 78.0±1.6b | 0.4 | -1.3 |
| Tb3 | 14.1±4.4a | 6.8 | 1.4 |  | 113.7±16.8a | 14.2 | 15.9 |  | 88.5±22.3bc | 3.3 | 4.6 |  | 77.3±13.3b | -9.8 | -10.3 |  | 27.93±12.18a | 2.3 | 6.6 |  | 76.7±1.6c | -1.3 | -2.9 |
| Tb4 | 13.7±4.3a | 3.8 | -1.4 |  | 118.7±17.6a | 19.2 | 21.0 |  | 98.8±15.5a | 15.3 | 16.8 |  | 83.4±6.3a | -2.7 | -3.2 |  | 28.95±9.59a | 6.0 | 10.5 |  | 76.9±1.5c | -1.0 | -2.7 |

^a^ Number with different letters are significantly different at *P* < 0.05 based on Duncan’s multiple range tests (NPB, 40 plants; WT and mutational lines, 20 plants)

^b^ D1, increase over NPB (%)

^c^ D2, increase over WT (%)
