# Supplementary material for: Identification through fine mapping and verification using CRISPR/Cas9-targeted mutagenesis for a minor QTL controlling grain weight in rice
Source: Theor Appl Genet. 2020 Oct 17;134(1):327–37. doi: 10.1007/s00122-020-03699-6 (PMC7813696; doi:10.1007/s00122-020-03699-6)
Supplement: Supplementary file 2 — Supplementary file2 (PDF 161 kb) [file 122_2020_3699_MOESM2_ESM.pdf]

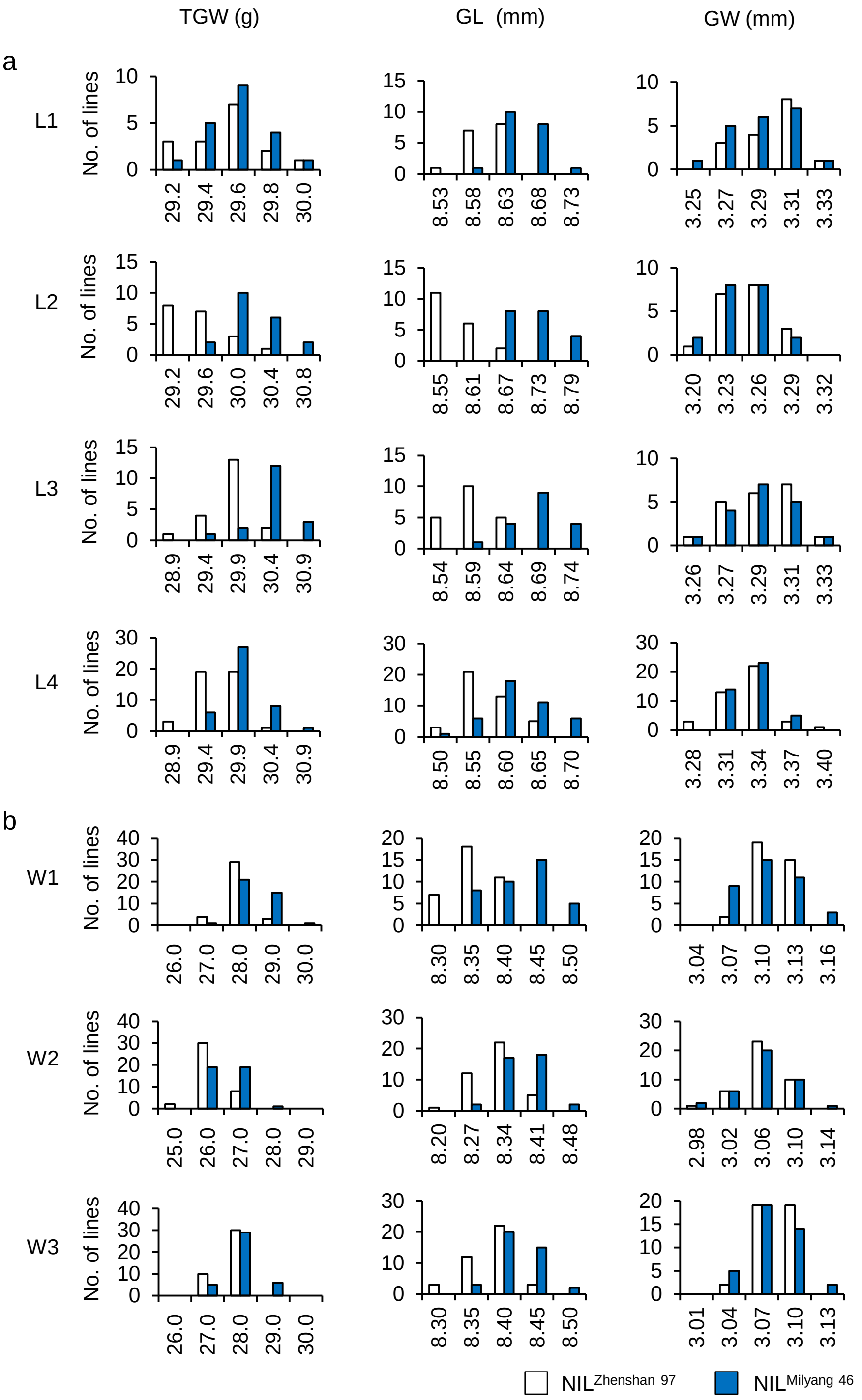

Fig. S1 Distribution of 1000-grain weight (TGW), grain length (GL) and grain width (GW) in the near isogenic lines (NILs). **a** The four NIL populations in $BC_2F_{11:12}$ in 2014, **b** The three NIL populations in $BC_2F_{14:15}$ in 2016.

```
ZS97  ATGGGCAACTGCTGTCCTGGCTCTGGGGATGCGGAGCCCGCCTCCTCCGACGCCTCCACCGGCAACGGCTCGTCCTCCTTCAAGGCCGGC  90
MY46  ATGGGCAACTGCTGTCCTGGCTCTGGGGATGCGGAGCCCGCCTCCTCCGACGCCTCCACCGGCAACGGCTCGTCCTCCTTCAAGGCCGGC  90

ZS97  GCGTCGCCGTCGTCGGCGCCCGCGCAGAACAAGCCGCCGGCGCCCATCGGGCCCGTGCTCGGCCGCCCCATGGAGGACGTCCGGAGCATC 180
MY46  GCGTCGCCGTCGTCGGCGCCCGCGCAGAACAAGCCGCCGGCGCCCATCGGGCCCGTGCTCGGCCGCCCCATGGAGGACGTCCGGAGCATC 180

ZS97  TACACCATCGGCAAGGAGCTCGGGCGCGGGCAGTTCGGCGTCACCTCCCTGTGCACCCACAAGGCGACGGGGCAGAAGTTCGCGTGCAAG 270
MY46  TACACCATCGGCAAGGAGCTCGGGCGCGGGCAGTTCGGCGTCACCTCCCTGTGCACCCACAAGGCGACGGGGCAGAAGTTCGCGTGCAAG 270

ZS97  ACCATCGCCAAGCGGAAGCTGTCCACCAAGGAGGACGTGGAGGACGTGCGGCGGGAGGTGCAGATCATGTACCACCTCGCCGGGCAGCCC 360
MY46  ACCATCGCCAAGCGGAAGCTGTCCACCAAGGAGGACGTGGAGGACGTGCGGCGGGAGGTGCAGATCATGTACCACCTCGCCGGGCAGCCC 360

ZS97  AACGTGGTGGAGCTCAAGGGCGCCTACGAGGACAAGCAGTCCGTCCACCTCGTCATGGAGCTCTGCGCCGGCGGCGAGCTCTTCGACCGG 450
MY46  AACGTGGTGGAGCTCAAGGGCGCCTACGAGGACAAGCAGTCCGTCCACCTCGTCATGGAGCTCTGCGCCGGCGGCGAGCTCTTCGACCGG 450

ZS97  ATCATCGCCAAGGGCCACTACACCGAGCGCGCCGCCGCCTCGCTCCTCCGCACCATCGTCGAGATCATCCACACCTGCCACTCCCTCGGC 540
MY46  ATCATCGCCAAGGGCCACTACACCGAGCGCGCCGCCGCCTCGCTCCTCCGCACCATCGTCGAGATCATCCACACCTGCCACTCCCTCGGC 540

ZS97  GTCATCCACCGCGACCTCAAGCCCGAGAACTTCCTCCTCCTCAGCAAGGACGAGGACGCCCCTCTCAAGGCCACCGACTTCGGCCTCTCC 630
MY46  GTCATCCACCGCGACCTCAAGCCCGAGAACTTCCTCCTCCTCAGCAAGGACGAGGACGCCCCTCTCAAGGCCACCGACTTCGGCCTCTCC 630
                                                                                        700
ZS97  GTCTTCTTCAAGCAAGGCACGCACACCTCCATTGAACTCTTTCCTTCCTCTCCTCAATGGCGCATCGACCTGAATTGAATTCATCCGTTG 720
MY46  GTCTTCTTCAAGCAAGGCACGCACACCTCCATTGAACTCTTTCCTTCCTCTCCTCAATGGCGCATCGACTTGAATTGAATTCATCCGTTG 720

ZS97  CCGCCATTGTTGATTGCAGGAGAGGTGTTCAAGGACATCGTCGGCAGCGCGTACTACATCGCGCCGGAGGTGCTGAAGCGGAGCTACGGC 810
MY46  CCGCCATTGTTGATTGCAGGAGAGGTGTTCAAGGACATCGTCGGCAGCGCGTACTACATCGCGCCGGAGGTGCTGAAGCGGAGCTACGGC 810

ZS97  CCCGAGGCGGACATCTGGAGCGTCGGCGTCATCCTCTACATCCTCCTCTGCGGTGTTCCTCCATTCTGGGCTGGCAAGTCATCTTGATCT 900
MY46  CCCGAGGCGGACATCTGGAGCGTCGGCGTCATCCTCTACATCCTCCTCTGCGGTGTTCCTCCATTCTGGGCTGGCAAGTCATCTTGATCT 900
                                                 945
ZS97  TTCCTCCCATCCATTTCGTCGAACACATCAACGGCGATGTGATGACTGGAGGAGTTAATCGATCTCTCATGTTTCAGAATCGGAGCATGG 990
MY46  TTCCTCCCATCCATTTCGTCGAACACATCAACGGCGATGTGATGGCTGGAGGAGTTAATCGATCTCTCATGTTTCAGAATCGGAGCATGG 990

ZS97  CATCTTCAATTCCATCCTGAGAGGGCAGGTGGACTTCACCAGTGACCCATGGCCACGCATTTCAGCTAGCGCCAAGGACCTTGTCAGGAA 1080
MY46  CATCTTCAATTCCATCCTGAGAGGGCAGGTGGACTTCACCAGTGACCCATGGCCACGCATTTCAGCTAGCGCCAAGGACCTTGTCAGGAA 1080

ZS97  GATGCTCAACTCCGACCCCAAGAAGAGAATTTCAGCCTACGAAGTCCTCAGTACGTGCCTCTTCTGGTTCTCCATTTTGCATCGTTTCAT 1170
MY46  GATGCTCAACTCCGACCCCAAGAAGAGAATTTCAGCCTACGAAGTCCTCAGTACGTGCCTCTTCTGGTTCTCCATTTTGCATCGTTTCAT 1170

ZS97  TTGGGATGTTTGAAATTTTAACTAACAATGCAATTTTTGGTTGTTAATTAGATCACCCGTGGATCAAGGAAGATGGAGAAGCACCTGACA 1260
MY46  TTGGGATGTTTGAAATTTTAACTAACAATGCAATTTTTGGTTGTTAATTAGATCACCCGTGGATCAAGGAAGATGGAGAAGCACCTGACA 1260

ZS97  CGCCGCTTGACAACGCTGTCATGAACAGGCTCAAGCAGTTCAGGGCTATGAACCAATTCAAGAAAGCAGCGCTAAGGGTCTGTGATTTTG 1350
MY46  CGCCGCTTGACAACGCTGTCATGAACAGGCTCAAGCAGTTCAGGGCTATGAACCAATTCAAGAAAGCAGCGCTAAGGGTCTGTGATTTTG 1350
                                   1381                                                  1438
ZS97  TTTCCCCTTTCAGCAACTACACTTCTGATCTACGAACAACCCACCTCAGCTTCTTGTTTCTCACTGAATCCGTTGGACAAAAAAAAATAT 1440
MY46  TTTCCCCTTTCAGCAACTACACTTCTGATCCACGAACAACCCACCTCAGCTTCTTGTTTCTCACTGAATCCGTTGGACAAAAAAAAA-AT 1440

ZS97  AAATTAAAAGGTCATTGCTGGATGCTTGTCGGAGGAAGAGATCAGAGGGCTGAAGGAGATGTTCAAGAGCATGGACTCTGACAACAGCGG 1530
MY46  AAATTAAAAGGTCATTGCTGGATGCTTGTCGGAGGAAGAGATCAGAGGGCTGAAGGAGATGTTCAAGAGCATGGACTCTGACAACAGCGG 1530

ZS97  CACCATTACCGTCGATGAGCTGCGCAAAGGGCTGTCCAAGCAAGGGACCAAGCTCACGGAGGCCGAAGTGCAGCAGCTAATGGAGGCTGT 1620
MY46  CACCATTACCGTCGATGAGCTGCGCAAAGGGCTGTCCAAGCAAGGGACCAAGCTCACGGAGGCCGAAGTGCAGCAGCTAATGGAGGCTGT 1620

ZS97  AAGTGTGCACACTCTCTTGGGGGCTTTGGCTTTTCTTTGTATCCGAGTTGTGATCTGCTACATGGTATAGGTGCAATGCTTAATCACTGC 1710
MY46  AAGTGTGCACACTCTCTTGGGGGCTTTGGCTTTTCTTTGTATCCGAGTTGTGATCTGCTACATGGTATAGGTGCAATGCTTAATCACTGC 1710
                1725
ZS97  AATGTTAAATTTTGTTGCGAAATATTCAGGCCGACGCGGACGGGAACGGAACCATCGATTACGACGAGTTCATCACAGCGACGATGCACA 1800
MY46  AATGTTAAATTTTGCTGCGAAATATTCAGGCCGACGCGGACGGGAACGGAACCATCGATTACGACGAGTTCATCACAGCGACGATGCACA 1800

ZS97  TGAACAGGATGGACAGAGAGGAGCATCTTTACACCGCGTTTCAGTATTTCGACAAGGACAACAGCGGGTAACTACTGCAGATTCTCCAAA 1890
MY46  TGAACAGGATGGACAGAGAGGAGCATCTTTACACCGCGTTTCAGTATTTCGACAAGGACAACAGCGGGTAACTACTGCAGATTCTCCAAA 1890
           1900                                       1938
ZS97  ATTTGATGC-AAAAATTACACAAGAGAGCAGTCAAATTCTTTATTTAAGATCTGTGCGTGTACCTCTGCAGCTGCATTTCGAAGGAGGAG 1980
MY46  ATTTGATGCAAAAAATTACACAAGAGAGCAGTCAAATTCTTTATTTATGATCTGTGCGTGTACCTCTGCAGCTGCATTTCGAAGGAGGAG 1980

ZS97  CTCGAGCAAGCGCTGAGGGAGAAAGGTCTTCTCGATGGCAGAGACATCAAAGACATCATATCAGAAGTCGACGCCGATAACGTAAAATCG 2070
MY46  CTCGAGCAAGCGCTGAGGGAGAAAGGTCTTCTCGATGGCAGAGACATCAAAGACATCATATCAGAAGTCGACGCCGATAACGTAAAATCG 2070
                                                       2127
ZS97  CAGCAGATTCCTTTGCTCTACAAATCATTTCTAACATTCTACAACACGAGTGACACGGCAATGCCAATGATCAATGTAGAGCTGACTAAT 2160
MY46  CAGCAGATTCCTTTGCTCTACAAATCATTTCTAACATTCTACAACACGAGTGACAAGGCAATGCCAATGATCAATGTAGAGCTGACTAAT 2160

ZS97  TTCGCTGGAATTTTACCTGCAGGATGGAAGGATAGACTACAGCGAGTTCGCGGCGATGATGCGGAAGGGAAACCCGGAGGCCAATCCCAA 2250
MY46  TTCGCTGGAATTTTACCTGCAGGATGGAAGGATAGACTACAGCGAGTTCGCGGCGATGATGCGGAAGGGAAACCCGGAGGCCAATCCCAA 2250

ZS97  GAAGCGACGGGACGTTGTGATATAG 2275
MY46  GAAGCGACGGGACGTTGTGATATAG 2275
```

Fig. S2 DNA sequence alignment of *OsCDPK2* between Zhenshan 97 (ZS97) and Milyang 46 (MY46). Introns are highlighted using gray background. Polymorphic events are highlighted using red letter, and their positions are shown by the number above.

```
ZS97  ATGGATCGGCAGAGGCAGCAGAGCTCCAGGGGCAATGCTACTGCAACGAGGGGTGGTGGGTCGTCGGGGAAGGGTGGTGGTGGTGGTGTC  90
MY46  ATGGATCGGCAGAGGCAGCAGAGCTCCAGGGGCAATGCTACTGCAACGAGGGGTGGTGGGTCGTCGGGGAAGGGTGGTGGTGGTGGTGTC  90

ZS97  GGGAAGGCGGCGGGGAAGAAGCCGATCAAGGTGGTGTACATCTCCAACCCCATGCGGGTCAAGACCAGCGCCGCCGGGTTCCGCGCCCTC 180
MY46  GGGAAGGCGGCGGGGAAGAAGCCGATCAAGGTGGTGTACATCTCCAACCCCATGCGGGTCAAGACCAGCGCCGCCGGGTTCCGCGCCCTC 180
                                                                                259
ZS97  GTGCAGGAGCTCACCGGCCGCAACGCCGACCCTTCCAAGTACAGCCCCCGCGCCTCCGCCGACGACGACGACGGCGGCGGCGGCGGCGGC 270
MY46  GTGCAGGAGCTCACCGGCCGCAACGCCGACCCTTCCAAGTACAGCCCCCGCGCCTCCGCCGACGACGACGACGGCGGCAGCGGCGGCGGC 270
                                     306
ZS97  GGCGGCGAGCTGGCCGCCGCCAGTGACGGCGCGGGAGAGCCCGGGCCCGGCGCCGCCGCGGCCTCGCCCGACACCGGCGCCGCAGCCGCC 360
MY46  GGCGGCGAGCTGGCCGCCGCCAGTGACGGCGCGGGCGAGCCCGGGCCCGGCGCCGCCGCGGCCTCGCCCGACACCGGCGCCGCAGCCGCC 360

ZS97  AGCGACGCCGCCGACGCCCTCGTGGCGGCGGGTCATCCGGCGGCGGCGACGTTCGACGACGAAGGCGGCGGTGGCGGCGGGGGATACTAC 450
MY46  AGCGACGCCGCCGACGCCCTCGTGGCGGCGGGTCATCCGGCGGCGGCGACGTTCGACGACGAAGGCGGCGGTGGCGGCGGGGGATACTAC 450
                   469-471                                                       528
ZS97  GACGACGACGACGACGAC---ATCTTCAGGTCGCAGCTGCTGGACACCAGCTACTCGGTGTTCTCGCCGCCGACGCTGCTCTACGACCAC 537
MY46  GACGACGACGACGACGACGACATCTTCAGGTCGCAGCTGCTGGACACCAGCTACTCGGTGTTCTCGCCGCCGACGCTGCTTTACGACCAC 540


ZS97  CCGCACAGCAAGGTGTAG      555
MY46  CCGCACAGCAAGGTGTAG      558
```

Fig. S3 DNA sequence alignment of *OsVQ4* between Zhenshan 97 (ZS97) and Milyang 46 (MY46) on the only exon of *OsVQ4*. Polymorphic events are highlighted using yellow backgroud, and their positions are shown by the number above.

```
                                                                 57       66                  86
ZS97 MDRQRQQSSRGNATATRGGGSSGKGGGGGVGKAAGKKPIKVVYISNPMRVKTSAAGFRALVQELTGRNADPSKYSPRASADDDDGGGGGGGGE  93
MY46 MDRQRQQSSRGNATATRGGGSSGKGGGGGVGKAAGKKPIKVVYISNPMRVKTSAAGFRALVQELTGRNADPSKYSPRASADDDDGSGGGGGGE  93
NPB  MDRQRQQSSRGNATATRGGGSSGKGGGGGVGKAAGKKPIKVVYISNPMRVKTSAAGFRALVQELTGRNADPSKYSPRASADDDDGGGGGGGGE  93
WT   MDRQRQQSSRGNATATRGGGSSGKGGGGGVGKAAGKKPIKVVYISNPMRVKTSAAGFRALVQELTGRNADPSKYSPRASADDDDGGGGGGGGE  93
Tb1  MDRQRQQSSRGNATANEGWWVVGEGWWWWCREGGGEEADQGGVHLQPHAGQDQRRRVPRPRAGAHRPQRRPFQVQPPRLRRRRRRRRRRRRRR  93
Tb2  MDRQRQQSSRGNATANEGWWVVGEGWWWWCREGGGEEADQGGVHLQPHAGQDQRRRVPRPRAGAHRPQRRPFQVQPPRLRRRRRRRRRRRRRR  93
Tb3  MDRQRQQSSRGNATATSGWWVVGEGWWWWCREGGGEEADQGGVHLQPHAGQDQRRRVPRPRAGAHRPQRRPFQVQPPRLRRRRRRRRRRRRRR  93
Tb4  MDRQRQQSSRGNATATSGWWVVGEGWWWWCREGGGEEADQGGVHLQPHAGQDQRRRVPRPRAGAHRPQRRPFQVQPPRLRRRRRRRRRRRRRR  93

                                                                   157
ZS97 LAAASDGAGEPGPGAAAASPDTGAAAASDAADALVAAGHPAAATFDDEGGGGGGGYYDDDDDD-IFRSQLLDTSYSVFSPPTLLYDHPHSKV   184
MY46 LAAASDGAGEPGPGAAAASPDTGAAAASDAADALVAAGHPAAATFDDEGGGGGGGYYDDDDDDDIFRSQLLDTSYSVFSPPTLLYDHPHSKV   185
NPB  LAAASDGAGEPGPGAAAASPDTGAAAASDAADALVAAGHPAAATFDDEGGGGGGGYYDDDDDD-IFRSQLLDTSYSVFSPPTLLYDHPHSKV   184
WT   LAAASDGAGEPGPGAAAASPDTGAAAASDAADALVAAGHPAAATFDDEGGGGGGGYYDDDDDD-IFRSQLLDTSYSVFSPPTLLYDHPHSKV   184
Tb1  AGRRQ  98
Tb2  AGRRQ  98
Tb3  AGRRQ  98
Tb4  AGRRQ  98
```

Fig. S4 Amino acid sequences of OsVQ4 in Zhenshan 97 (ZS97), Milyang 46(MY46), Nipponbare (NPB), the wild-type transgenic line (WT) and the four mutational lines of *OsVQ4*. Sequences reserved in the mutational lines are highlighted in red letter. The VQ-motif is highlighted in green background. Differences between ZS97 and MY46 are highlighted in yellow background. The positions of the motif and differences are shown by the number above.

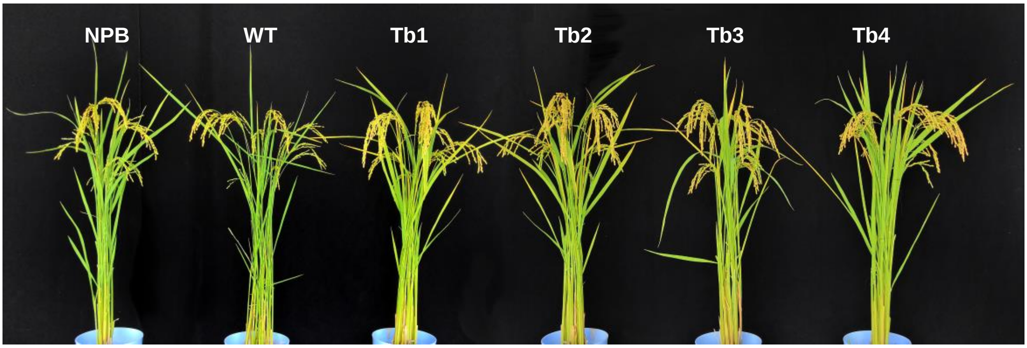

Fig. S5 Plants of Nipponbare (NPB), the wild-type transgenic line (WT) and the four mutational lines of *OsVQ4*.
